# Antiapoptotic BCL-2 proteins determine sorafenib/regorafenib resistance and BH3-mimetic efficacy in hepatocellular carcinoma

**DOI:** 10.18632/oncotarget.24673

**Published:** 2018-03-30

**Authors:** Anna Tutusaus, Milica Stefanovic, Loreto Boix, Blanca Cucarull, Aynara Zamora, Laura Blasco, Pablo García de Frutos, Maria Reig, Jose C. Fernandez-Checa, Montserrat Marí, Anna Colell, Jordi Bruix, Albert Morales

**Affiliations:** ^1^ Department of Cell Death and Proliferation, IIBB-CSIC, IDIBAPS, Barcelona, Spain; ^2^ Departament de Biomedicina, Facultat de Medicina, Universitat de Barcelona, Barcelona, Spain; ^3^ Barcelona Clinic Liver Cancer Group, Liver Unit, Hospital Clínic of Barcelona, University of Barcelona, CIBEREHD, IDIBAPS, Barcelona, Spain; ^4^ Liver Unit, Hospital Clinic, CIBEREHD, Barcelona, Spain; ^5^ Research Center for Alcoholic Liver and Pancreatic Diseases, Keck School of Medicine of the University of Southern California, Los Angeles, CA, USA

**Keywords:** liver cancer, BCL-2 family proteins, BH3-mimetics, mitochondria/apoptosis, sorafenib

## Abstract

Sorafenib, systemic treatment for advanced hepatocellular carcinoma (HCC), and regorafenib, novel second line treatment after sorafenib failure, have efficacy limited by evasive mechanisms of acquired-drug resistance. BCL-2 proteins participate in the response to tyrosine kinase inhibitors; however, their role in HCC therapy with sorafenib/regorafenib remains uncertain. BH3-mimetic ABT-263 (navitoclax) enhanced sorafenib activity, inducing cell death via a mitochondrial caspase-dependent mechanism, after BCL-xL/BCL-2 inhibition. Sorafenib-resistant hepatoma cells (HepG2R and Hep3BR) exhibited altered mRNA expression of BCL-2 and other anti-apoptotic family members, such as MCL-1, priming drug-resistant cancer cells to death by BH3-mimetics. ABT-263 restored sorafenib efficacy in sorafenib-resistant cell lines and HCC mouse models. Moreover, in mice xenografts from patient-derived BCLC9 cells, better tumor response to sorafenib was associated to higher changes in the BCL-2 mRNA pattern. HCC non-treated patients displayed altered BCL-2, MCL-1 and BCL-xL mRNA levels respect to adjacent non-tumoral biopsies and an increased BCL-2/MCL-1 ratio, predictive of navitoclax efficacy. Moreover, regorafenib administration also modified the BCL-2/MCL-1 ratio and navitoclax sensitized hepatoma cells to regorafenib by a mitochondrial caspase-dependent mechanism. In conclusion, sorafenib/regorafenib response is determined by BCL-2 proteins, while increased BCL-2/MCL-1 ratio in HCC sensitizes drug resistant-tumors against ABT-263 co-administration. Thus, changes in the BCL-2 profile, altered in HCC patients, could help to follow-up sorafenib efficacy, allowing patient selection for combined therapy with BH3-mimetics or early switch them to second line therapy.

## INTRODUCTION

Hepatocellular carcinoma (HCC), the most common liver cancer [1], is often diagnosed at an advanced stage with poor prognosis. Its expected incidence increases due to the growing prevalence of non-alcoholic fatty liver disease associated to obesity and metabolic syndrome [2]. Despite several key therapeutic advancements the sole systemic agents with survival benefit are the multikinase inhibitors sorafenib [3] and regorafenib [4]. Unfortunately, the activity of these drugs is limited by primary and acquired drug resistance mechanisms [5, 6]. Thus, there is need to develop additional therapeutic agents to further enhance the still limited survival of the patients. The mechanisms for sorafenib and regorafenib escape are not well known. The role of cell death-related pathways involving mitochondria is gaining interest as an alternative approach to target cancers where the dependence on specific driver mutations for survival is not established. In this sense, HCC is a tumor with a complex genetic background where clear druggable addictions have not been validated.

The BCL-2 family proteins control apoptosis at the mitochondria by regulating mitochondrial outer membrane permeabilization (MOMP) by multidomain proapoptotic BAX and BAK. MOMP results in the cytosolic release of proapoptotic mitochondrial intermembrane space proteins such as cytochrome c and smac/DIABLO, leading to caspase activation and rapid cell death [7, 8]. Given this pivotal role, MOMP is highly regulated, mostly by pro- and anti-apoptotic members of the BCL-2 protein family. Among them, some BH3-only proteins directly activate BAX and BAK, such as BID, BIM, and PUMA, while others inactivate the multidomain anti-apoptotic BCL-2, BCL-XL, and MCL-1, such as BAD and NOXA. Multiple insults favor the MOMP and change the delicate balance established between activators and repressors of BAX/BAK homo-oligomerization, resulting in cell death or in the appearance of drug resistance. BCL-2 family proteins and the mitochondrial-dependent apoptotic program have been previously reported as main components of the sorafenib cytotoxicity in hepatoma cells [9–14]. MCL-1 depletion and BAX mitochondrial translocation [9, 11], BAD increase [12], BIM status [13], mitochondrial oxidative stress accumulation [11, 14], are frequently observed after sorafenib exposure. Increased BCL-2 family members, particularly BCL-xL levels have been connected to HCC growth [15] and sorafenib-resistance [16], validating the mitochondrial pathway as highly relevant in sorafenib therapy for HCC. This link was further demonstrated by the increased antitumoral activity registered when combining the BH3-mimetic ABT-737 with sorafenib [13, 17].

Specific BCL-2 profiles that sensitize or provide therapeutic resistance against particular chemotherapeutic agents have been recently identified [18–22]. Novel strategies, based on ectopic expression and genetic/pharmacological repression of specific BCL-2 proteins [23, 24], “mito-prime” cancer cells against drug treatments [25]. However, neither a specific signature in BCL-2 proteins has been associated to sorafenib response, nor mitochondrial “priming for death” defined after sorafenib exposure. Interestingly, several BH3-mimetics that interact with specific BCL-2 family proteins have been identified and studied in on-going clinical trials. Consequently, we aimed to evaluate the occurrence of BCL-2 addiction in the sorafenib resistance displayed by HCC tumors, and to determine whether BH3-mimetics could take advantage of this mitochondrial adaptation during sorafenib treatment.

## RESULTS

### ABT-263 potentiates sorafenib activity on hepatoma cell lines

**Figure 1:**
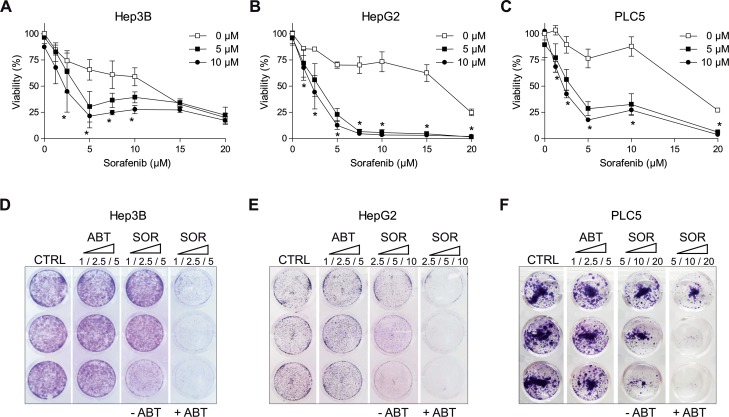
ABT-263 potentiated sorafenib cytotoxicity against different hepatoma cell lines **(A-C)** MTT assays to test ABT-263 effect on sorafenib cytotoxicity in different liver cell lines (Hep3B, HepG2 and PLC5). **(D-F)** Crystal Violet staining was performed after 3 days exposure of sorafenib/ABT-263 in Hep3B, HepG2 and PLC5 cell cultures. (n=3) ^*^P< 0.05 vs. control.

Our previous work [11] prompted our interest in searching therapeutic strategies that benefit from sorafenib cytotoxic effects in mitochondria. Taking advantage of this mitochondrial sensitization, BH3-mimetics could interact with anti-apoptotic BCL-2 family members to increase sorafenib efficacy in hepatoma cells. Hep3B, HepG2 and PLC5 cells were treated with navitoclax (ABT-263) a specific BCL-2 and BCL-xL inhibitor in clinical study and exposed to sorafenib to test their efficacy (Figure 1A–1C). ABT-263 potentiated the cytotoxic sorafenib effect in all hepatoma cell lines tested (Figure 1A–1C). To verify that navitoclax was our best option, we tested other reported BH3-mimetics and, although all compounds increased cell death to some extent, ABT-263 was clearly more efficient in combination with sorafenib on hepatoma cells (Supplementary Figure 1). After three days exposure, Hep3B, HepG2 and PLC5 cells treated with navitoclax were visibly sensitized to sorafenib (Figure 1D–1F), reproducing in crystal violet assays the MTT findings. This combined activity, in line with previous observations [16, 26], evidenced that ABT-263 is a potent sensitizer for sorafenib action in HCC cell lines.

**Figure 2:**
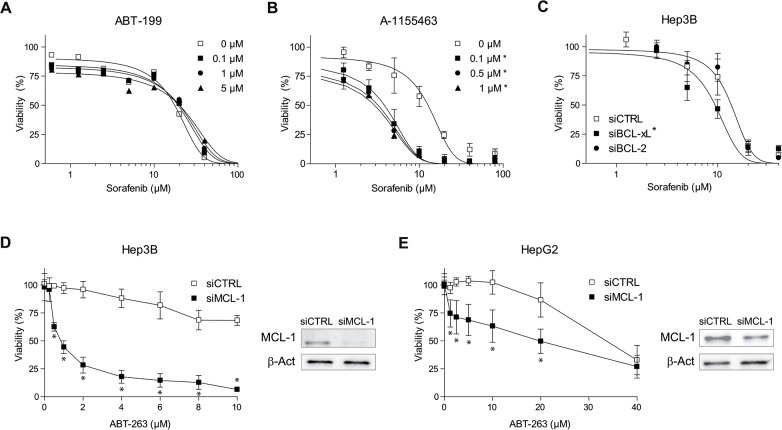
Sorafenib and ABT-263 activity on hepatoma cells depend on anti-apoptotic BCL-2 protein levels **(A** and **B)** Cell viability of Hep3B cells treated with specific inhibitors ABT-199 (BCL-2) and A-1155463 (BCL-xL) were tested in combination with sorafenib (μM). **(C)** Cell viability of Hep3B cells transfected with siRNAs control and against BCL-2 or BCL-xL, and treated with sorafenib. RNA interference was validated by qPCR. ^*^P< 0.05 vs. control or siCTRL cells. **(D** and **E)** Hep3B and HepG2 cells were transfected with siRNAs control and against MCL-1 and treated and MTT assays performed after ABT-263 exposure. In parallel panels, protein levels of MCL-1 and β-actin are showed. (n=3) ^*^P< 0.05 vs. control or siCTRL cells

Since ABT-263 effect on sorafenib efficacy is expected due to its sequestration of intracellular BCL-2 and BCL-xL, we analyzed the effect of separately targeting both proteins. First, the FDA-approved BCL-2 inhibitor ABT-199, even when used at high doses (0.1-5 μM), was unable to potentiate sorafenib activity in Hep3B cells (Figure 2A) or in HepG2 cells (Supplementary Figure 2). In contrast, the potent and selective BCL-xL inhibitor A-1155463 exhibited similar efficacy than the observed after navitoclax administration (Figure 2B). Supporting these observations, BCL-xL mRNA reduction (72±5% knockdown), via siRNA transfection in Hep3B cells, recapitulated the increased sorafenib effect induced by navitoclax (Figure 2C). However, BCL-2 reduction (64±10% knockdown) did not significantly sensitize against sorafenib, while co-silencing BCL-2/BCL-xL was equally effective (not shown here).

Therefore, the well-known decrease of MCL-1 induced by sorafenib [11, 16], combined to BCL-xL/BCL-2 reduction by RNA silencing or ABT-263 treatment, could be enough to cause hepatoma cell death as previously reported [16, 26]. Confirming this hypothesis, MCL-1 targeting was highly effective to kill Hep3B and HepG2 cells exposed to ABT-263 (Figure 2D, E). In sum, these experiments illustrate the ability of individual changes in BCL-2 family proteins to modulate sorafenib efficacy in hepatoma cells.

### BCL-2, MCL1 and BCL-xL mRNA levels are altered in tumoral tissue from HCC patients

**Figure 3:**
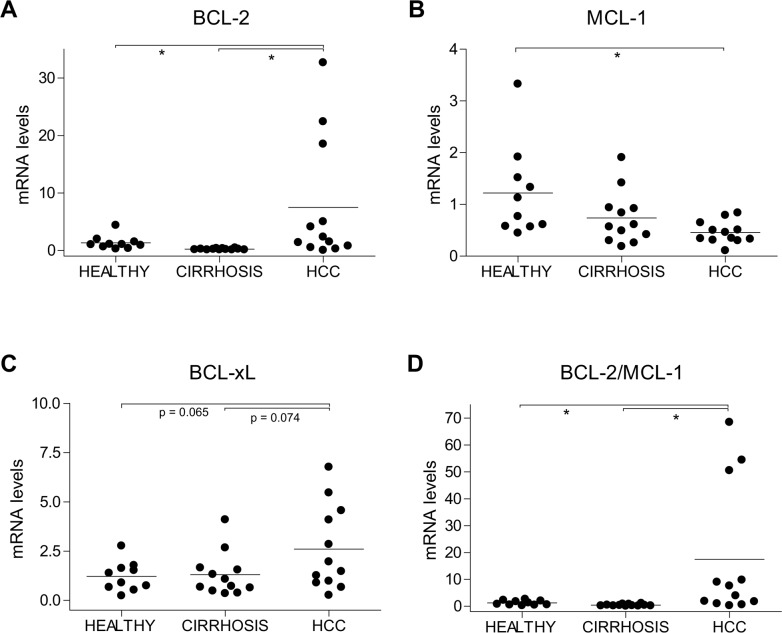
Alterations in BCL-2, MCL-1 and BCL-xL mRNA levels in HCC patients **(A)** BCL-2, **(B)** MCL-1, **(C)** BCL-xL and **(D)** BCL-2/MCL-1 mRNA levels were measured by qPCR in healthy liver (n=10) and in cirrhotic and tumoral tissue from HCC patients (n=12) with HCV/EtOH etiology. ^*^P< 0.05.

At this point, it would be interesting to analyze in human biopsies BCL-2, MCL-1 or BCL-xL levels during follow-up to test their correlation with the tumor response under sorafenib. Unfortunately, sorafenib treatment is delivered to patients with advanced hepatocellular carcinoma that are not routinely biopsied just prior to treatment. To gain some insight into the expression pattern of BCL-2 family protein, we tested in untreated HCC samples (<5cm) without vascular invasion (I-II TNM stage) from Ethanol/HCV cirrhotic patients (n=12) for mRNA changes respect to adjacent non-tumoral biopsies (n=12) and to healthy livers (n=10), as detailed in Supplementary Figure 3. HCC biopsies exhibited enhanced BCL-2 and decreased MCL-1 levels compared to control livers (Figure 3A, B). In addition, some individuals exhibited increased BCL-xL levels in cirrhotic or tumoral areas (Figure 3C). The BCL-2/MCL-1 ratio has been proposed as predictor of *in vitro* sensitivity to navitoclax in human myeloma cell lines [22]. Interestingly, a significant increase in BCL-2/MCL-1 was displayed by HCC samples, that was not presented by the neighboring cirrhotic tissue (Figure 3D), suggesting that this modification could be associated to tumor development.

### BCL-2 protein alteration during sorafenib resistance renders hepatoma cells sensitive to ABT-263 administration

**Figure 4:**
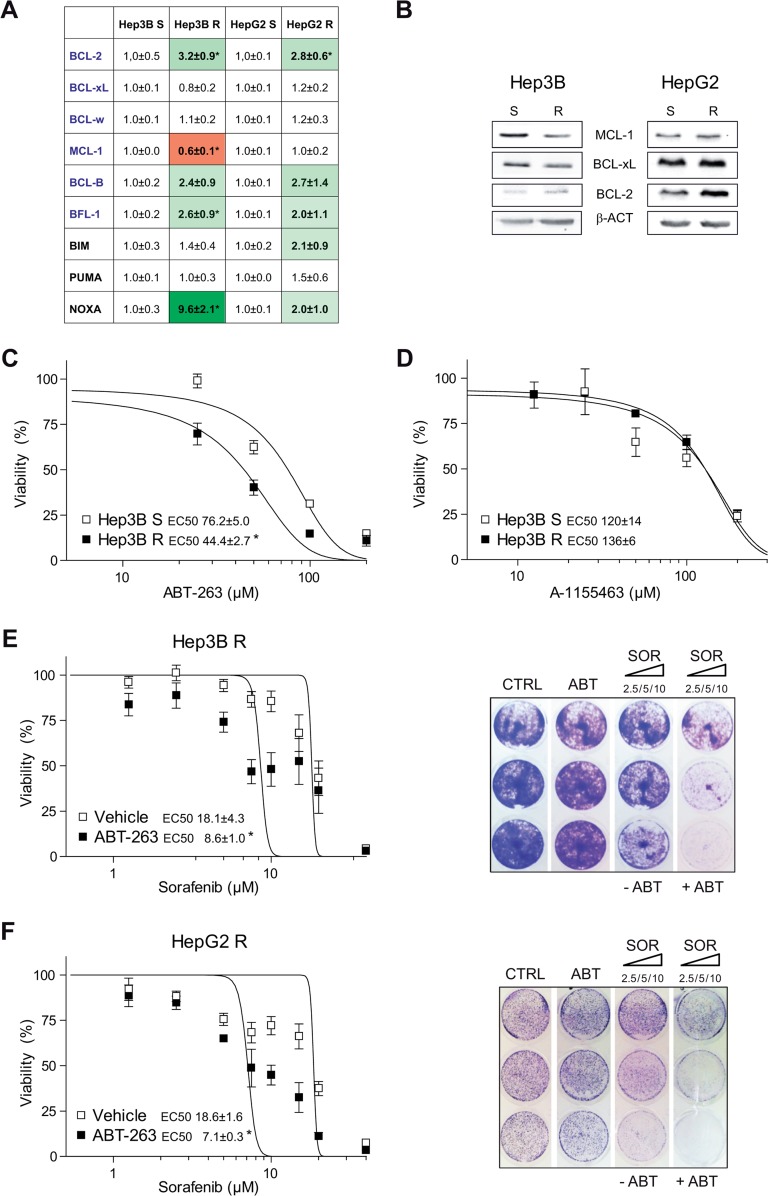
Sorafenib-resistant hepatoma cells exhibit mRNA changes in BCL-2 proteins and are re-sensitized to sorafenib by ABT-263 exposure **(A)** mRNAs levels of different BCL-2 family proteins from sorafenib sensitive (S) and sorafenib resistant (R) Hep3B and HepG2 cells. ^*^P< 0.05 vs. sensitive hepatoma cells. **(B)** Representative western blot images of MCL-1, BCL-xL, BCL-2 and β-Actin protein levels exhibited by Hep3B and HepG2 sorafenib sensitive (S) and sorafenib resistant (R) cells. **(C)** Effect of ABT-263 in sensitive and sorafenib-resistant Hep3B cells. ^*^P< 0.05 vs. sensitive cells. **(D)** Effect of A-1155463 exposure to sensitive and sorafenib-resistant Hep3B cells. **(E-F)** Effect of ABT-263 administration on sorafenib response in sorafenib-resistant Hep3B and HepG2 cells, measured by MTT assay. ^*^P< 0.05 vs. vehicle-treated cells. Representative Crystal violet staining images. (n=3).

After long-time exposure to sorafenib, we analyzed in sorafenib-resistant HepG2 and Hep3B cells if mRNA changes in anti-apoptotic BCL-2 related proteins could be induced by sorafenib resistance, and provide clues about potential BH3-mimetic uses. A significant BCL-2 increase was observed in both resistant cell lines, respect to their parental cells (Figure 4A). Particularly, an enhanced BCL-2/MCL-1 ratio (5.4±0.9 and 2.9±0.7, respectively) was detected. Minor changes in BCL-2/MCL-1 protein levels were detected in HepG2 R and Hep3B R (2.2±0.4 and 1.4±0.3) by western blot (Figure 4B), suggesting mRNA analysis as more sensitive indicator of anti-apoptotic BCL-2 modifications in our samples.

In addition, changes in pro-apoptotic BCL-2 family members were also detected in resistant cell lines. Particularly in Hep3B cells, an important alteration in several BCL-2-related mRNAs was caused by drug-exposure. Analogous alterations in the BCL-2 network, recently observed in other cellular settings [23], have been postulated to create a BCL-2 addiction used by BH3-mimetics to eliminate tumor cells. In agreement, sorafenib-resistant cells Hep3B were sensitive to ABT-263 alone (Figure 4C), while BCL-xL inhibitor A-1155463 did not kill resistant hepatoma cells preferentially, as observed in Hep3B R (Figure 4D) and HepG2 R cells (Supplementary Figure 4).

Furthermore, navitoclax overcame sorafenib resistance in both hepatoma cell lines (Figure 4E, F), while BCL-2 inhibitor ABT-199 did not (Supplementary Figure 5). These results indicate that, although changes in BCL-xL have not been observed in HCC tumors or after sorafenib-exposure, basal BCL-xL levels should be playing an important role in hepatoma cells. Similarly sensitization by navitoclax was observed in crystal violet assays, underscoring the requirement for the simultaneous BCL-2/BCL-xL inhibition to counteract sorafenib-resistance.

### ABT-263 triggered mitochondrial-dependent apoptotic cell death after sorafenib exposure in hepatoma cell lines

**Figure 5:**
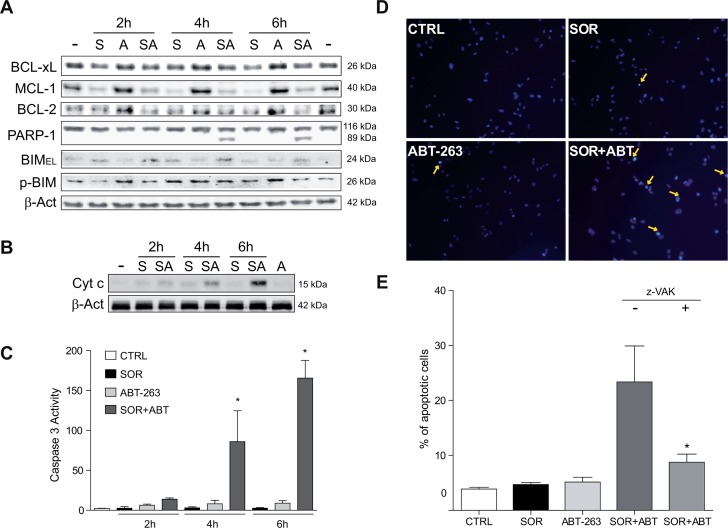
Sorafenib/ABT-263 combination induced apoptotic cell death via a mitochondrial caspase-dependent mechanism **(A)** Hep3B cells were exposed to sorafenib (S, 10 μM) with or without ABT-263 (A, 5 μM) and expression levels of indicated proteins were analyzed by Western blot, using β-actin as a loading control. **(B)** cytochrome c levels were analyzed by western blot in the cytosol of Hep3B cells at different times, and **(C)** fold increase in caspase-3 activity was determined in total cell extracts as above. **(D-E)** Nuclear Hoechst 33258 staining was visualized in Hep3B cells treated with sorafenib and/or ABT-263, and apoptotic positive cells counted in 10 independent fields per condition. (n=3) ^*^P< 0.05 vs. control Hep3B cells.

ABT-263 is a potent BH3-mimetic that selectively inhibits BCL-xL and BCL-2. Thus, we expected that sorafenib-induced mitochondrial membrane permeabilization, and consequent release of intermembrane proteins such as cytochrome c, could be accelerated by ABT-263. Among main anti-apoptotic BCL-2 proteins, only MCL-1 was obviously affected by short-time sorafenib exposure, while no evident changes in BCL-2 or BCL-xL were detected (Figure 5A). In parallel, an increased in pro-apoptotic BIM levels was induced in sorafenib-treated samples, probably due to the minor phospho-BIM levels, and consequent proteosomal degradation, caused by sorafenib. Interestingly, PARP-1 cleavage was early identified suggesting that ABT-263 initiated cell death in sorafenib treated cells following MCL-1 levels depletion and BIM increase.

Similar to the timing of PARP-1 degradation, Hep3B cells pretreated with navitoclax exhibited a quick mitochondrial release of cytochrome c (Figure 5B), (Figure 5B) while no cytosolic increase in this apoptogenic protein was detected after 2-6 hours of sorafenib exposure. Similar patterns of cytochrome c and rapid PARP-1 cleavage after ABT-263 incubation were observed in sorafenib-treated HepG2 and PLC5 cells, (Supplementary Figure 6). Incidentally, no additional reduction in mitochondrial membrane potential or ATP levels were noticed in sorafenib-treated cells after navitoclax administration (Supplementary Figure 7).

Sorafenib is an inducer of autophagy, which could preserve survival after cytochrome c release in the absence of caspase activation [27]. To verify that the MOMP was leading to apoptotic cell death by a caspase-dependent mechanism, we measured caspase-3 activity. A remarkable increase in caspase-3 activity was detected following ABT-263 addition to sorafenib-treated hepatoma cells (Figure 5C), paralleling the PARP proteolysis previously observed. Consistent with an apoptotic sequence of events, an evident nuclear condensation and fragmentation was visualized in sorafenib-treated cells incubated with navitoclax (Figure 5D). Of note, the pre-addition of a pancaspase inhibitor Z-VAD-FMK (z-VAK) significantly reduced the amount of apoptotic Hep3B cells (8.5±2.0 vs. 24.5±4.1) counted after the ABT-263/sorafenib combination (Figure 5E).

### Sorafenib modifies the BCL-2 system in HCC mouse models and benefits from ABT-263/sorafenib co-administration

**Figure 6:**
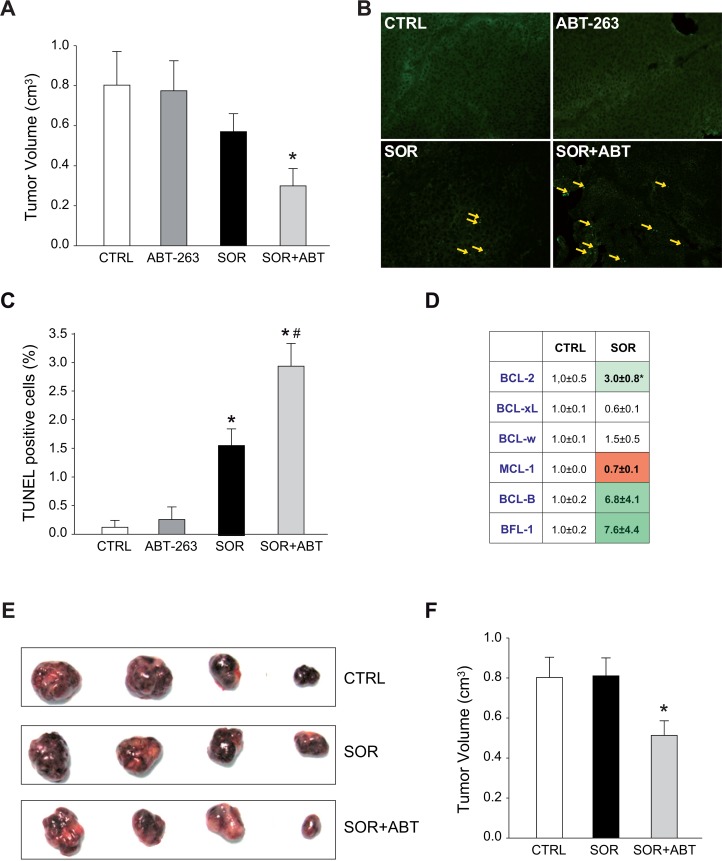
Sorafenib altered the mRNA BCL-2 pattern *in vivo* and ABT-263 increases sorafenib efficacy in murine subcutaneous HepG2 models **(A)** Tumor growth in mice bearing HepG2-subcutaneous tumors that were given orally ABT-263 (100 mg/kg) and/or sorafenib (80 mg/kg) daily for 3 weeks (CTRL, n=6; ABT, n=3; SOR, n=6; SOR+ABT, n=8). ^*^P< 0.05 vs. vehicle-treated mice. **(B-C)** Representative images of tumor samples stained for TUNEL detection and graphic quantification. **(D)** Representation of anti-apoptotic BCL-2 mRNAs levels in HepG2 tumors treated with vehicle or sorafenib. Subcutaneous tumor samples were obtained from mice gavaged with vehicle (CTRL) or sorafenib (SOR) daily for 3 weeks. ^*^P< 0.05 vs. vehicle-treated tumors. **(E-F)** Representative images and tumor growth quantification of sorafenib-resistant HepG2 tumors in mice treated orally with sorafenib (80 mg/kg) and ABT-263 (100 mg/kg) for 3 weeks (n=6). ^*^P< 0.05 vs. vehicle-treated mice.

HepG2 cells were subcutaneously inoculated in nude mice and treated with sorafenib, ABT-263 or the combination of both drugs. HepG2 tumors treated with ABT-263/sorafenib exhibited minor tumor growth (Figure 6A) and increased cell death, as visualized by TUNEL staining (Figure 6B), and quantified (Figure 6C). The anti-apoptotic BCL-2 protein system was analyzed in sorafenib-treated tumors. Enhanced mRNA levels of BCL-2 (3.0±0.8) and BCL-2/MCL-1 (3.4±0.7) ratio compared to vehicle-tumors (Figure 6D) were detected; supporting that sorafenib exposure is triggering a BCL-2 profile alteration. Accordingly, when injecting sorafenib-resistant HepG2 cell subcutaneously to mice, the tumors recovered sensitivity to sorafenib exposure when the animals were gavaged simultaneously with sorafenib and ABT-263 (Figure 6E, 6F). Neither sorafenib nor navitoclax alone (data not shown) reduced tumor growth.

### Sorafenib alters the antiapoptotic BCL-2 profile predicting tumor response *in vivo*

**Figure 7:**
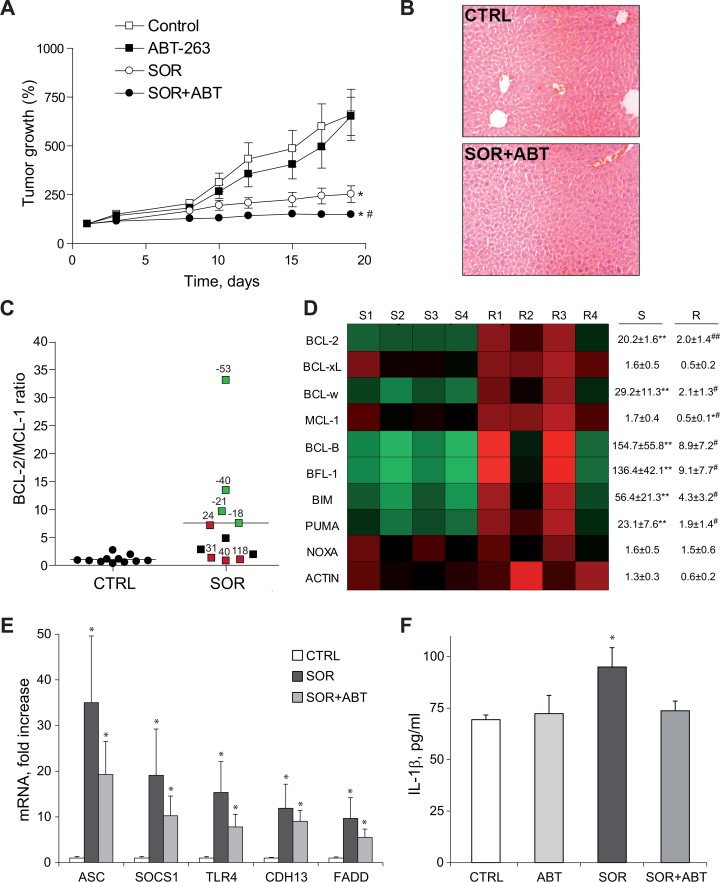
ABT-263 potentiated sorafenib effectiveness in an experimental HCC model, being changes in the BCL-2 profile indicative of sorafenib response **(A)** Tumor growth in mice bearing BCLC9-subcutaneous tumors that were daily gavaged with ABT-263 (100 mg/kg) and/or sorafenib (80 mg/kg) (CTRL, n=12; ABT, n=6; SOR, n=11; SOR+ABT, n=8). ^*^P< 0.05 vs. vehicle-treated mice. *^#^P<* 0.05 vs. sorafenib-treated mice. **(B)** Representative H&E liver images from vehicle and sorafenib/ABT-263 treated mice. **(C)** BCL-2/MCL-1 mRNA ratio of BCLC9 tumors treated with vehicle (CTRL) or sorafenib (SOR). Samples in green exhibited increased sorafenib efficacy, while tumors in red were less responsive (% respect to median growth). **(D)** Representation of mRNA changes in members of BCL-2 family exhibited by sensitive (S1 to S4) and resistant (R1 to R4) BCLC9 tumors following sorafenib administration. ^*^P< 0.05, ^**^P< 0.01 vs. vehicle-treated tumors. *^#^P<* 0.05, *^##^P<* 0.01 vs. sorafenib-sensitive tumors. **(E)** mRNA levels of ASC, SOCS1, TLR4, CDH13 and FADD in tumors from mice treated with vehicle, sorafenib or sorafenib/ABT-263 (n=4). **(F)** Serum levels of human IL-1β in mice as above. ^*^P< 0.05 vs. vehicle-treated mice.

To verify these observations we used the anchor-free growing human hepatocellular carcinoma cells. BCLC9 cells, established from a well-differentiated human HCC, exhibit a stem cell phenotype and are highly efficient tumor initiating cells in nude mice [28]. Mimicking the results obtained in mice bearing HepG2 tumors, ABT-263 was highly effective reducing the growth of BCLC9 tumors treated with sorafenib (Figure 7A). No hepatic damage was caused, as denoted by lack of variations in serum ALT/AST levels (data not shown) and H&E staining of liver biopsies (Figure 7B).

Once again, an altered BCL-2 protein pattern was observed upon sorafenib exposure. Interestingly, when we analyzed the BCL-2/MCL-1 ratio, predictive value of the observed ABT-263 efficacy (Figure 7C), we distinguished a parallelism between tumor growth and BCL-2/MCL-1 levels among sorafenib-only treated tumors (n=11). BCLC9 tumors more sensitive to sorafenib (green squares) displayed higher BCL-2/MCL-1 ratios compared to more resistant tumors (red squares) where sorafenib was less effective (Figure 7C). Sorafenib-treated BCLC9 tumors not only up-regulated anti-apoptotic BCL-2 proteins such as BCL-2 or BCL-w, but also increased other pro-apoptotic BCL-2 members such as BIM, with higher values associated to better sorafenib response (Figure 7D). In addition, a good correlation was found between BCL-2/MCL-1 expression and sorafenib-induced cytotoxicity (0.938, Pearson coefficient) in the hepatoma cell lines examined (Supplementary Figure 8), maybe supporting this ratio also as indicative of sorafenib efficacy.

### Sorafenib-induced inflammation is reduced by navitoclax co-administration

Finally, we analyzed the modification of cellular pathways involved in liver cancer in sorafenib-treated BCLC9 tumors using a gene array. When we focused on mRNAs presenting major upregulation following sorafenib exposure, we detected an increase in genes associated to inflammasome activation and inflammatory response such as TLR4 and ASC, among others (Figure 7E). As readout of inflammasome induction we checked for serum IL-1β levels in the treated animals. IL-1β was significantly increased in the serum of BCLC9-bearing mice treated with sorafenib (Figure 7F). This relationship inflammation/tumor growth is consistent with the observation that inflammatory side effects related to sorafenib administration, such as dermatology hand-food-skin reaction, are associated to slower tumor growth and improved survival. Interestingly, ABT co-administration potentiated cell death and reduced tumor growth without additional inflammatory reaction. On the contrary, decreased IL-1β circulatory levels were exhibited by mice receiving sorafenib/ABT-263 suggesting the possibility of achieving increase sorafenib activity, not linked to enhanced inflammation.

### Regorafenib modifies the BCL-2/MCL-1 ratio in hepatoma cells and ABT-263 co-addition induces mitochondrial caspase-dependent death

**Figure 8:**
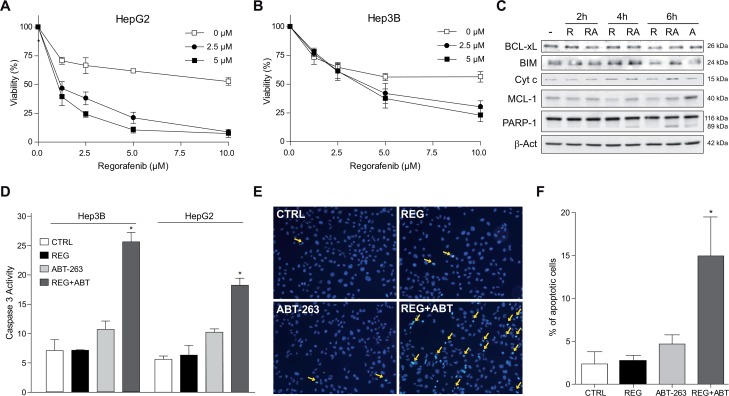
Regorafenib/ABT-263 combination induced apoptotic cell death via a mitochondrial caspase-dependent mechanism **(A-B)** HepG2 and Hep3B cells were exposed to regorafenib/ABT-263 and MTT viability measured after 16 h. **(C)** cytochrome c levels were analyzed by western blot in the cytosol and BCL-xL, BIM, MCL-1 and PARP-1 in total extracts from hepatoma cells treated with regorafenib (5 μM) and/or ABT-263 (5 μM). **(D)** fold increase in caspase-3 activity was determined in total cell extracts as above. **(E-F)** Nuclear Hoechst 33258 staining was visualized in Hep3B cells treated with regorafenib and/or ABT-263, and apoptotic positive cells counted in 10 independent fields per condition. (n=3) ^*^P< 0.05 vs. control cells.

Since regorafenib has been approved as second line treatment for HCC progressing after sorafenib, we analyzed if navitoclax could also potentiate regorafenib activity. First, we measured mRNA changes in the BCL-2/MCL-1 ratio. In HepG2 cells, regorafenib highly increased the BCL-2/MCL-1 levels after 10 hours (15.6±4.8 fold increase), while a 5.4±1.2 fold increase was observed in Hep3B cells. Consistently, ABT-263 greatly sensitized HepG2 cells to regorafenib (Figure 8A), and had significant effects on Hep3B cells (Figure 8B). As soon as 4 hours after regorafenib exposure, MCL-1 levels were reduced in HepG2 cells, probably due to an increase proteasomal degradation induced by regorafenib ERK inhibition [29], mechanism also relevant in sorafenib action as observed before. Moreover, ABT-263 co-administration induced cytosolic cytochrome c released and PARP-1 cleavage (Figure 8C), and a moderate increased in BIM levels was detected. Similar effects were observed in Hep3B cells (data not shown), inducing obvious apoptotic features as denoted by caspase-3 activity (Figure 8D), Hoechst staining (Figure 8E) and apoptotic cell counting (Figure 8F).

## DISCUSSION

Sorafenib, drug recommended as first-line of treatment for HCC patients [3, 30], requires assistance to completely halt HCC progression. Although immune checkpoint inhibition could be helpful [31], so far less than 25% of HCC patients exhibit a molecular signature that could be linked to objective response to immune-targeted therapy [32]. Therefore, we still need early markers of sorafenib efficacy or failure. Quantifying BCL-2 family members in sorafenib HCC therapy, aligns with proposed determinations of mRNA and protein BCL-2 profile [19–22, 33, 34] as indicative of treatment efficacy in colorectal cancer, lymphoid and myeloid leukemia, among others. Currently, profiling by tumor biopsy has no impact in HCC clinical decision-making and most patients are diagnosed by using imaging criteria [35]. Our work supports the analysis of BCL-2 family proteins in patients undergoing treatment, as potential tool to stratify them according to expected evolution under sorafenib.

The approval of regorafenib as second line drug after sorafenib treatment [4] underscores the clinical relevance of techniques to promptly switch patients from first line to second line therapy. Our *in vitro* data indicates that regorafenib also increases the BCL-2/MCL-1 ratio and ABT-263 administration is able to sensitize hepatoma cells against regorafenib. An altered BCL-2 pattern, present in HCC biopsies, may also be indicative of regorafenib response and of BH3-mimetics capacity to potentiate regorafenib activity. Besides immunotherapy, novel targets for sorafenib acquired resistance are proposed, such as Focal Adhesion Kinase [36], ceramide generation [11] or Metallothionein-1G [37]. In the absence of surrogate serum markers, biopsies of sorafenib-treated tumors will help to validate them, increasing our knowledge of HCC vulnerabilities.

**Figure 9:**
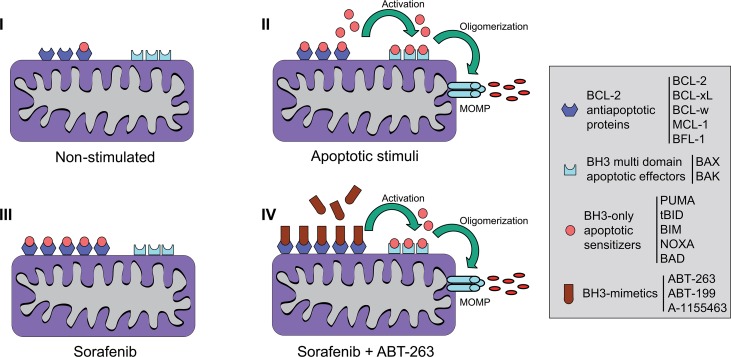
Scheme representing the mitochondrial effects induced by sorafenib and BH3-mimetics on hepatoma cells **(I)** Non-stimulated cells present expression of different BCL-2 family members. **(II)** After exposure to apoptotic stimuli that induce mitochondrial-dependent cell death, BH3-only apoptotic sensitizers, such as BIM or BID, if not sequestered by BCL-2 anti-apoptotic molecules, it will bind to and activate effector molecules BAX or BAK. This process leads to BAX/BAK oligomerization, mitochondrial membrane permeabilization, and the mitochondrial release of apoptogenic proteins such as cytochrome c. **(III)** Specific stimuli, like sorafenib, alter the levels of anti-apoptotic BCL-2 proteins avoiding cell death, but mito-priming the surviving cells to BH3-mimetics. **(IV)** In hepatoma cells after sorafenib exposure, ABT-263 sequesters anti-apoptotic BCL-2 and BCL-xL releasing BH3-only proteins such as BIM to bind BAX/BAK and trigger cell death.

Beyond the predictive value of BCL-2 profile in sorafenib/regorafenib efficacy, BH3 mimetics are interesting compounds in combination with sorafenib, since sorafenib resistance could be generating a targetable BCL-2 addiction (Figure 9). Most common mutations in HCC, such as p53 and beta-catenin, are undruggable. Therefore, new targetable proteins are required to improve HCC management. In this sense, several BH3 mimetics are already in clinical trials, and others, as BCL-xL inhibitors, are under scrutiny for cancer treatment as the biomedical importance of this pathway has been unveiled [38, 39]. Among them, venetoclax (ABT199) recently approved for leukemia therapy [40] and reported to cause minor side effects on treated patients appears highly promising [41]. Unfortunately, it does not seem to be effective in combination with sorafenib in HCC treatment (Figure 2A and Supplementary Figure 3). Although BCL-2/MCL-1 ratio could be an indicative marker of sorafenib activity, simultaneous BCL-xL inhibition seems to be required to potentiate sorafenib action. In agreement, navitoclax (ABT-263) is much more interesting, although the appearance of thrombocytopenia in patients has limited its development, particularly in hematologic malignancies [38]. In fact, an on-going clinical trial is testing the combination of navitoclax and sorafenib in HCC patients (NCT02143401), which would benefit from biomarkers for patient selection and follow up, such as the one we proposed.

Moreover, other BH3 mimetics could potentiate sorafenib effects. The lack of ABT-199 efficacy in combination with sorafenib, may suggest that HCC behaves as a BCL-xL-dependent tumor. So, BCL-xL inhibitors such as A-1155463 or A-133185 could deserve to be tested [35, 38, 39] in HCC treatment, despite not improving navitoclax effect in our models. As observed in patients, specific individuals exhibited remarkable differential expression of BCl-2 and BCL-xL that may justify different strategies in their treatments. Despite their similarities, BCL-2 and BCL-xL do not interact with the same proteins [39]. Both proteins are sensitized by increased BIM levels, as detected after sorafenib administration [42], but bind BAX and BAK differently. While all anti-apoptotic BCL-2 proteins bind BAX, only MCL-1 and BCL-xL bind BAK [43]. Coadministration of sorafenib and ABT-263 could unleash BAK from MCL-1 and BCL-xL respectively [44], and sequester BCL-2 from BAX, triggering simultaneously BAK activation and BAX translocation. Further studies could be necessary to assess this point, since complex interactions, and not simply expression patterns of BCL-2 proteins [45], may need to be investigated to optimize BH3-mimetics use and understand BCL-2 dependence. For instance, ABT-263 not also binds BCL-2 and BCL-xL but also increases the protein and mRNA levels of other BLC-2 proteins such as MCL-1 [46], besides activating ERK and AKT. Precisely through this kinase activity, navitoclax may also have an induction role in BCL-2 [47] and BCL-xL [48] expression. These effects exerted by ABT-263, and probably by other BH3-mimetics, should also be kept in mind. Furthermore, both navitoclax and the A-1331852 BCL-xL inhibitor induce apoptotic death in PDGF-activated hepatic fibroblast and *in vivo* reduce liver fibrosis in the MDR2^−/−^ mouse model [49], supporting a link between BH3-induced apoptosis and liver fibrogenesis [50]. It is tempting to speculate that these features may become a second benefit in liver cancer patients with underlying chronic liver disease.

Finally, clinical practice associates a better outcome in sorafenib therapy to the development of adverse events such as hand-food-skin reaction. Chemotherapy frequently induces inflammasome components that contribute to the body pro-inflammatory response [51, 52]. In particular, a relationship between dermatologic adverse events and complete response under sorafenib in patients with hepatocellular carcinoma has been recently established [53]. Probably related to this, sorafenib causes mitochondrial ROS production and a severe decline in mitochondrial membrane potential. Relevantly, recent reports associate inflammasome activation to mitochondrial injury [52–54] and inflammasome restriction to removal of damaged mitochondria [55]. Regarding this point, our data may not be sufficient to assess this complicated network, e.g. different inflammasomes, canonical vs. non-canonical activation. Yet, navitoclax effect on inflammasome induction and side-effects in patients are aspects that deserve further analysis.

In conclusion, the BCL-2 profile determines sorafenib/regorafenib response and could help to early stratify patients before and under treatment. Meanwhile, navitoclax in particular, and BH3-mimetics in general, reveal as an interesting sorafenib combination to overcome sorafenib resistance without additional inflammatory response.

## MATERIALS AND METHODS

### Reagents

Dulbecco's Modified Eagle's Medium (DMEM), trypsin-EDTA, penicillin-streptomycin and dimethyl sulfoxide (DMSO), MTT (3-(4,5-dimethylthiazol-2-yl)-2,5-diphenyl tetrazolium bromide) (M2128), Hoechst 33258 (B1155), Crystal Violet (C0755), and DCF (D6883) were purchased from Sigma-Aldrich (St. Louis, MO). All tissue culture-ware was from Nunc (Roskilde, Denmark). Biotin Blocking System, peroxidase substrate (DAB) and peroxidase buffer were from DAKO (Glostrup, Denmark). Aquatex was from Merck (Darmstadt, Germany). The ABC kit was from Vecstain (Burlingame, CA). Proteinase inhibitors were from Roche (Madrid, Spain). ECL western blotting substrate was from Pierce (Thermo Fisher Scientific, Rockford, IL). BCL-2 siRNA (h) (sc-29214), BCL-xL siRNA (h) (sc-43630), MCL-1 siRNA (h) (sc-35877) and scrambled controls were purchased from Santa Cruz Biotechnology (Dallas, TX). Lipofectamine2000 (11668-027), Novex Sharp Pre-stained Protein Standard (LC5800) and JC-1 (T-3168) were from Invitrogen Life Technologies (Carlsbad, CA). Sorafenib (BAY 43-9006, Nexavar) and Regorafenib (BAY 73-4506, Stivarga) are manufactured by Bayer. A-1155463, ABT-737, AT101, HA-14, ABT-263 (Navitoclax) and ABT-199 (Venetoclax) were purchased from Selleckchem (Houston, TX).

### Cell culture and biochemical analysis

Human liver tumor cell lines Hep3B, PLC and HepG2 (European Collection of Animal Cell Cultures (ECACC)) were grown in DMEM (10% FBS) at 37°C and 5% CO_2_. Sorafenib-resistant hepatoma cells [11], were maintained at 2.5 μM sorafenib and kept without drug at least one week before experiments. Cell viability measured by MTT assay and clonogenic assays by Crystal Violet Staining, Hoechst staining, and caspase-3 activity were analyzed as previously indicated [11, 56].

### Immunoblot analysis

Cell lysates were prepared in RIPA buffer plus proteinase inhibitors. Samples containing 10 to 30 μg were separated by 10-15% SDS-PAGE. Proteins were transferred to nitrocellulose membranes, blocked in 5% nonfat milk for 1h at room temperature, and incubated overnight at 4°C with the primary antibodies:

MCL-1 (S-19) (SantaCruz, sc-819) 1:250 rabbit

BCL-2 (C-2) (SantaCruz, sc-7382) 1:250 mouse

BCL-xL (H-5) (SantaCruz, sc-8392) 1:250 mouse

PARP-1 (H-250) (SantaCruz, sc-7150) 1:250 rabbit

BIM (H-191) (SantaCruz, sc-11425) 1:250 rabbit

Phospho-BIM (Ser69) (Cell Signaling, #4581) 1:1000 rabbit

Cytochrome C (SantaCruz, sc-1356) 1:250 mouse

β-Actin (Sigma-Aldrich, A3854) 1:40,000 conjugated to HRP

### RNA isolation and real time RT-PCR

Total RNA was isolated with TRIzol reagent. 1ug of RNA was reverse-transcribed with iScript™ cDNA Synthesis Kit (Biorad, Berkeley, CA) and real-time PCR was performed with iTaq™ Universal SYBR^®^ Green Supermix (Biorad, Berkeley, CA) following the manufacturer's instructions [11, 56]. The primers sequences used were:

**Table:** 

human BCL-2:	Fw 5′-GGAGGATTGTGGCCTTCTTT-3′ Rv 5′-GCCGTACAGTTCCACAAAGG-3′
human BCL-xL:	Fw 5′-GGATGGCCACTTACCTGA-3′ Rv 5′-CGGTTGAAGCGTTCCTG-3′
human MCL-1:	Fw 5′-ATGCTTCGGAAACTGGACAT-3′ Rv 5′-TCCTGATGCCACCTTCTAGG-3′
human BCL-B:	Fw 5′-GCTGGGATGGCTTTTGTCA-3′ Rv 5′-GCCTGGACCAGCTGTTTTCTC-3′
human BCL-w:	Fw 5′-ACCCCAGGCTCAGCCCAACA-3′ Rv 5′-CAGCACACAGTGCAGCCCCA-3′
human BFL-1:	Fw 5′-TTACAGGCTGGCTCAGGACT-3′ Rv 5′-AGCACTCTGGACGTTTTGCT-3′
human BIM:	Fw 5′-TGGCAAAGCAACCTTCTGATG-3′ Rv 5′-GCAGGCTGCAATTGTCTACCT-3′
human NOXA:	Fw 5′-TGGAAGTCGAGTGTGCTACTCA-3′ Rv 5′-CAGAAGAGTTTGGATATCAGATT-3′
human PUMA:	Fw 5′-GCATGCCTGCCTCACCTT-3′ Rv 5′-TCACACGTCGCTCTCTCTAAACC-3′
human β-Actin:	Fw 5′-AGAAAATCTGGCACCACACC-3′ Rv 5′-AGAGGCGTACAGGGATAGCA-3′
human 18S:	Fw 5′-CCGAAGATATGCTCATGTGG-3′ Rv 5′-TCTTGTACTGGCGTGGATTC-3′

### Immunohistochemical staining

Liver and tumor samples were fixed and 5-μm sections were prepared following standard procedures. The slices were examined with a Zeiss Axioplan microscope equipped with a Nikon DXM1200F digital camera. Apoptotic cells with fragmented nuclei were detected in paraffin samples using TUNEL labeling containing fluorescein-dUTP and -dNTPs (TUNEL Label Mix, Roche). TUNEL positive cells were observed and quantified using a NIKON Eclipse E-100 microscope.

### Tumor animal models

All animal procedures were performed according to protocols approved by the Animal Experimentation Ethics Committee from the University of Barcelona. For subcutaneous tumor model, male Swiss nude mice, 5-6 week old, were kept under pathogen-free conditions with free access to standard food and water. HepG2 cells (5×10^6^) or BCLC9 cells (2.5×10^6^) were injected subcutaneously into the flanks of mice in 100 μL DMEM without FBS, as previously reported [11, 57]. Treatments with ABT-263 (100 mg/Kg body weight), sorafenib (80 mg/Kg) or vehicle (12.5% Cremophor, 12.5% ethanol, 75% sterile saline) were delivered daily via oral gavage for 21 days. Tumors were measured periodically with a Vernier caliper, and the volume was calculated as length × width^2^ × 0.5.

### Gene array

A predesigned 384-well human Liver cancer panel (SAB Target List, H384 Cat#10034526) for SYBR Green detection (Bio-rad) was used following the manufacturer's instructions, as previously reported [56].

### HCC patient study

Samples, tumor and non-tumor biopsies, from twelve patients diagnosed with HCC and treated at the Clinic Hospital in Barcelona, and ten healthy liver samples from patients subjected to surgery due to colorectal cancer without any diagnosed liver disease, were included. Patients gave informed consent in according to the principles embodied in the Declaration of Helsinki.

### Statistical analyses

Results are expressed as mean ± standard deviation and n=3, unless indicated. Statistical comparisons were usually performed using unpaired 2-tailed Student's *t* test, and 1-way ANOVA followed by Newman-Keuls Multiple Comparison Test (GraphPad Prism) was used for data quantification from patients. A *P* value less than 0.05 was considered significant.

### Financial support

Study funded by grants from Instituto de Salud Carlos III (FIS PI15/00145 to M.R., FIS PI14/00962 to J.B., PI13/00374 and PI16/00930 to M.M., CIBEREHD and CIBERNED), Ministerio de Economía y Competitividad (SAF2015-69944-R to J.F.C., SAF2015-66515-R to A.M. and P.G.F., and SAF2013-47246-R to A.C.) and co-funded by FEDER (Fondo Europeo de Desarrollo Regional, Unión Europea); center grant P50-AA-11999 from Research Center for Liver and Pancreatic Diseases, US NIAAA to J.F.C.). Fundació Marató de TV3 (to A.C), AGAUR (2014_SGR_605 to J.B. and 2014_SGR_785 to J.F.C.) and CERCA Programme / Generalitat de Catalunya.

## SUPPLEMENTARY MATERIALS FIGURES



## Abbreviations

HCC: hepatocellular carcinoma
MMP: mitochondrial membrane potential
MOMP: mitochondrial outer membrane permeabilization
ROS: reactive oxygen species
WT: wild type
