# Supplementary material for: Antiapoptotic BCL-2 proteins determine sorafenib/regorafenib resistance and BH3-mimetic efficacy in hepatocellular carcinoma
Source: Oncotarget. 2018 Mar 30;9(24):16701–17. doi: 10.18632/oncotarget.24673 (PMC5908280; doi:10.18632/oncotarget.24673)
Supplement: Supplementary file 1 [file oncotarget-09-16701-s001.pdf]

Antiapoptotic BCL-2 proteins determine sorafenib/regorafenib resistance and BH3-mimetic efficacy in hepatocellular carcinoma

SUPPLEMENTARY MATERIALS

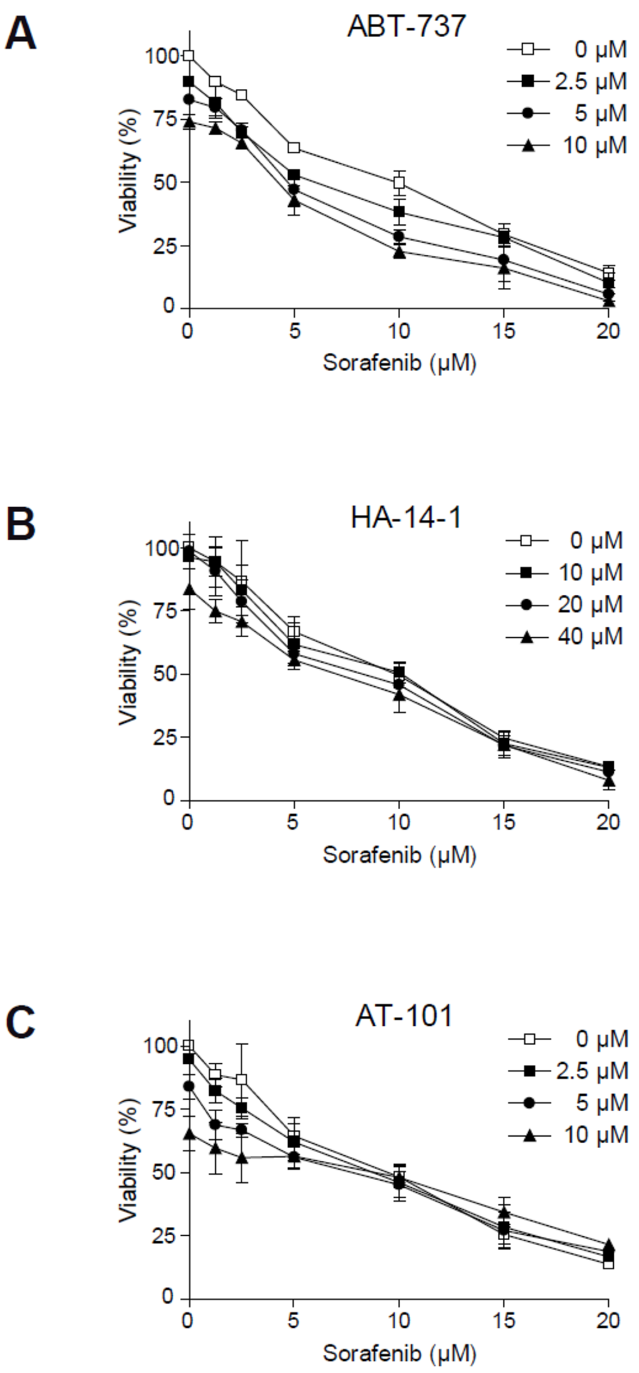

Supplementary Figure 1: Effect on sorafenib activity against hepatoma cells of different BH3 mimetics. MTT assays at different concentrations (μM) of compounds that interact with BCL-2 proteins, such as ABT-737 **(A)**, HA-14-1 **(B)** and AT-101 **(C)**, were tested in combination with sorafenib in Hep3B cells after 16 hours exposure (n=3).

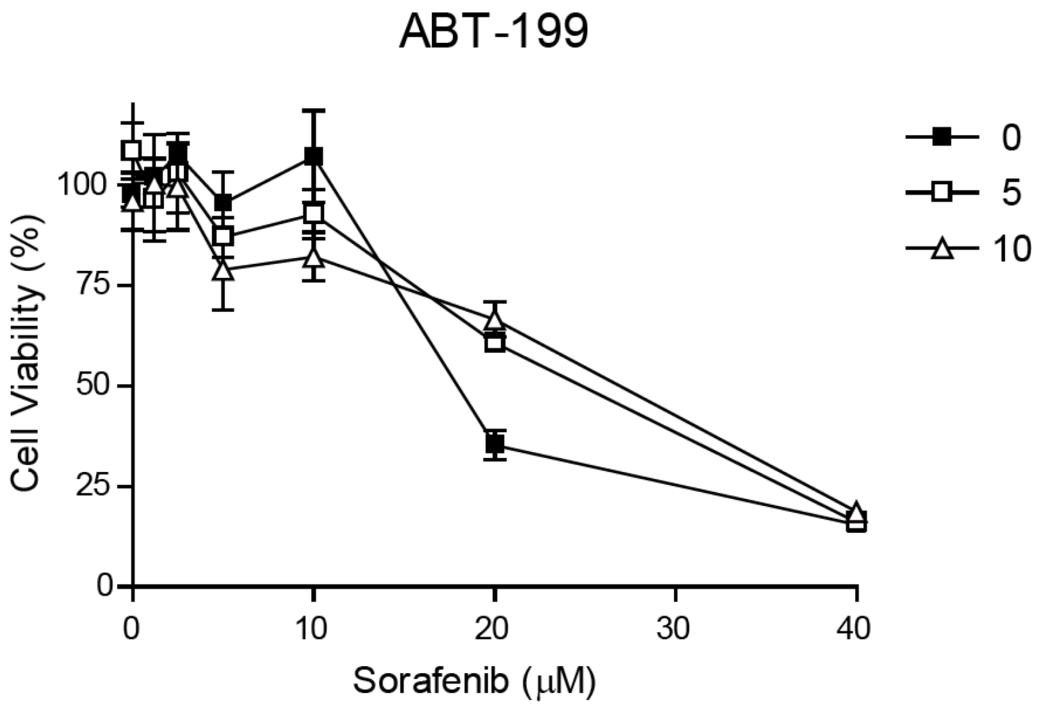

Supplementary Figure 2: BCL-2 inhibition alone (ABT-199) did not sensitize HepG2 cells against sorafenib. Cell viability after sorafenib administration in HepG2 cells treated with vehicle (0) or ABT-199 at 5 and 10 μM during 16 hours. (n=3).

GENDER	AGE	Underlying liver disease	ETHIOLOGY	Virus hepatitis	AFP (ngr/mL)	TUMOR SIZE (cm)	Tumor	Differentiation degree	Vascular Invasion
M	67	cirrhosis	alcohol	NO		4,5	single	NA	NA
M	61	cirrhosis	alcohol	NO	2	3,5	single + 1 satelite	well	No
M	47	cirrhosis	VHC	VHC	6	2,5	single		No
M	75	cirrhosis	VHC	VHC	23	1,1 (main tumor)	binodular	well	No
M	78	cirrhosis	VHC	VHC		4,5	single	well+moderate	No
M	73	cirrhosis	VHC	VHC	2	2,1	single	moderate	No
M	67	cirrhosis	VHC	VHC	22	2,1	single	well+moderate	No
M	54	cirrhosis	VHC+VHB+alcohol	VHC+VHB	15	1,3	single	well	NA
M	63	cirrhosis	VHC	VHC	2	2,2	single	well+moderate	No
M	64	cirrhosis	alcohol	NO	6	4,5	single	well	No
M	50	cirrhosis	VHC+alcohol	VHC	19	1,4	single	well	NA
F	61	cirrhosis	VHC	VHC	11	2,1	single	well	No
M	68	healthy liver	NA	NO	NA	NA	NA	NA	NA
F	70	healthy liver	NA	NO	NA	NA	NA	NA	NA
F	47	healthy liver	NA	NO	NA	NA	NA	NA	NA
F	50	healthy liver	NA	NO	NA	NA	NA	NA	NA
F	55	healthy liver	NA	NO	NA	NA	NA	NA	NA
M	36	healthy liver	NA	NO	NA	NA	NA	NA	NA
M	36	healthy liver	NA	NO	NA	NA	NA	NA	NA
M	55	healthy liver	NA	NO	NA	NA	NA	NA	NA
M	37	healthy liver	NA	NO	NA	NA	NA	NA	NA
F	73	healthy liver	NA	NO	NA	NA	NA	NA	NA

Supplementary Figure 3: Characteristics of patients included in the study.

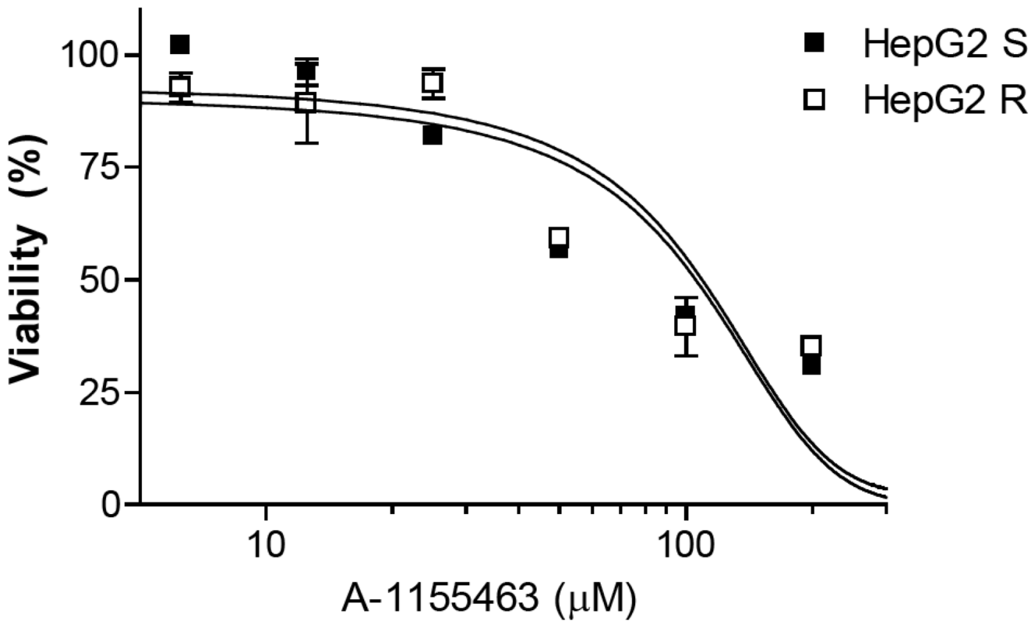

Supplementary Figure 4: Sorafenib-resistant HepG2 cells are not sensitized against BCL-xL inhibitor A-1155463. Cell viability after A-1155463 administration to sorafenib resistant (R) and sensitive (S) HepG2 cells during 16 hours. (n=3).

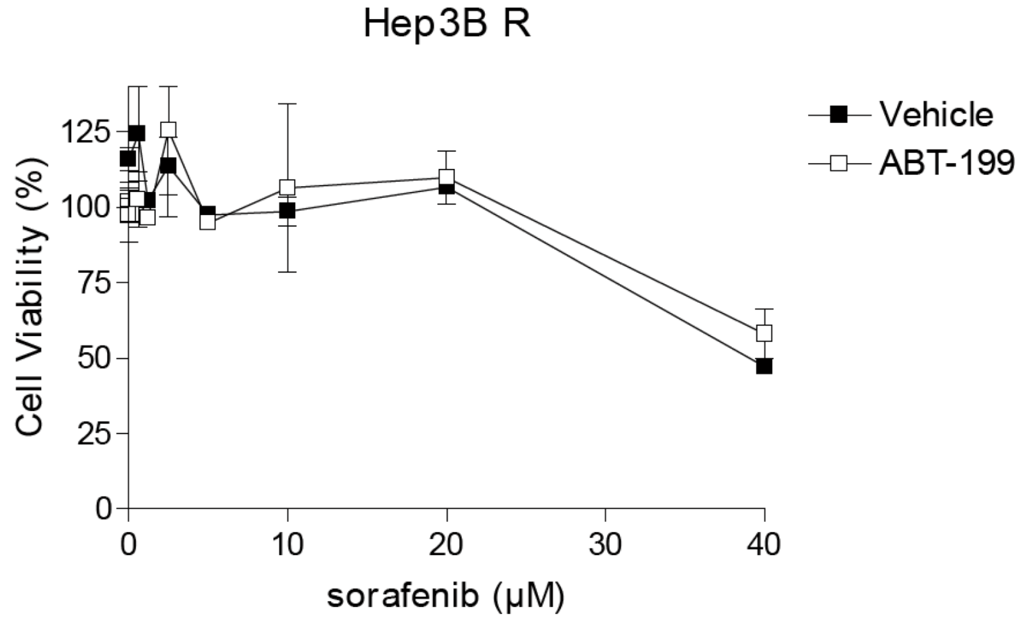

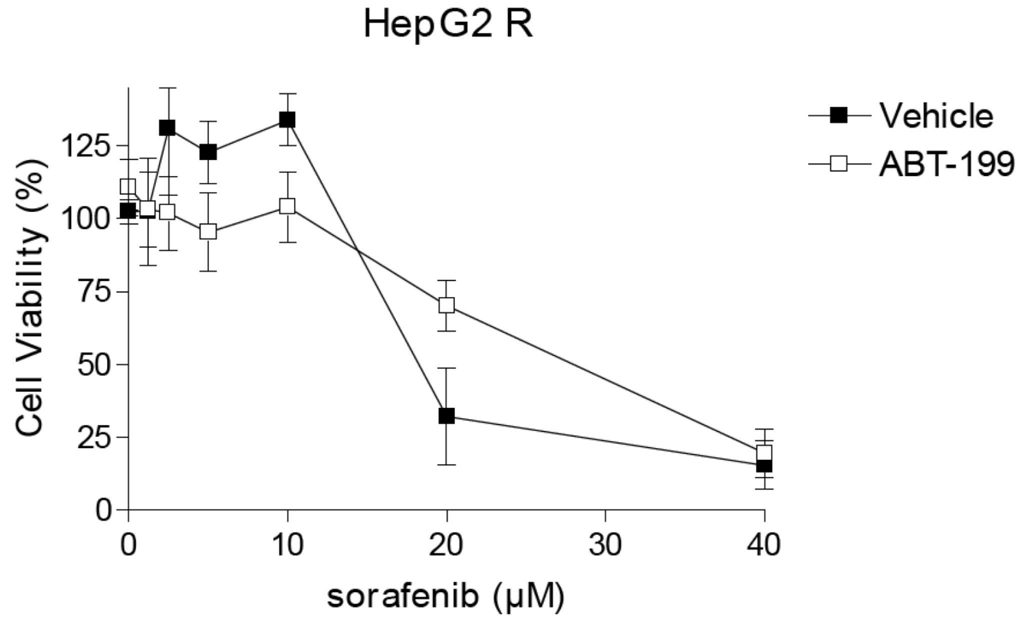

Supplementary Figure 5: BCL-2 inhibition alone (ABT-199) did not sensitize resistant hepatoma cells against sorafenib. Cell viability after sorafenib administration in HepG2 cells treated with vehicle or ABT-199 at 10 µM concentration during 16 hours. (n=3).

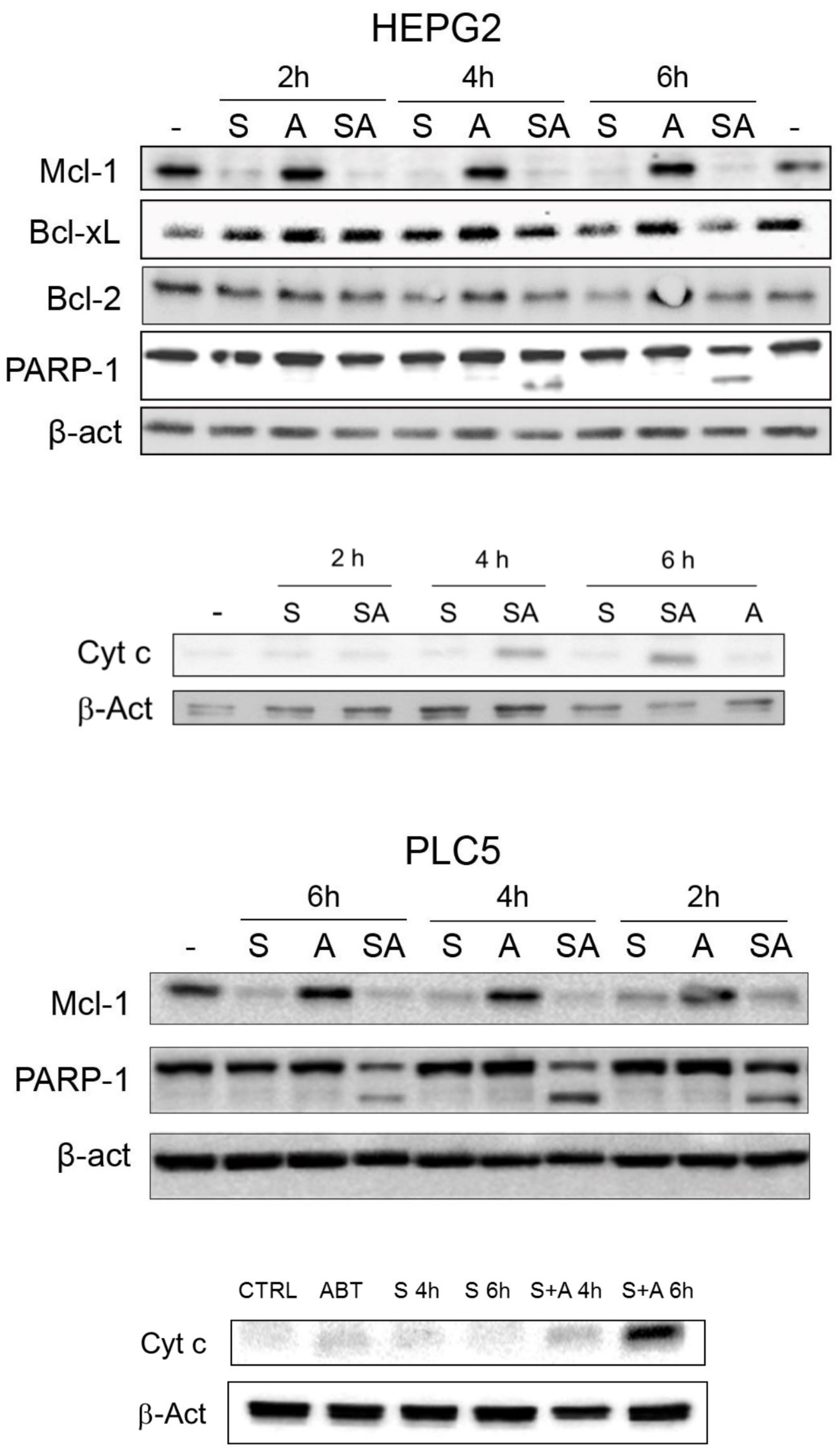

Supplementary Figure 6: ABT-263 induced mitochondrial dependent cell death in sorafenib-treated HepG2 and PLC5 cells. HepG2 and PLC5 cells were treated with sorafenib (S, 10 μM), ABT-263 (A, 5 μM) or the combination (SA) and analyzed at different times BCL-2 proteins, PARP-1 degradation and cytochrome c mitochondrial release (Cyt c), using β-actin as loading control.

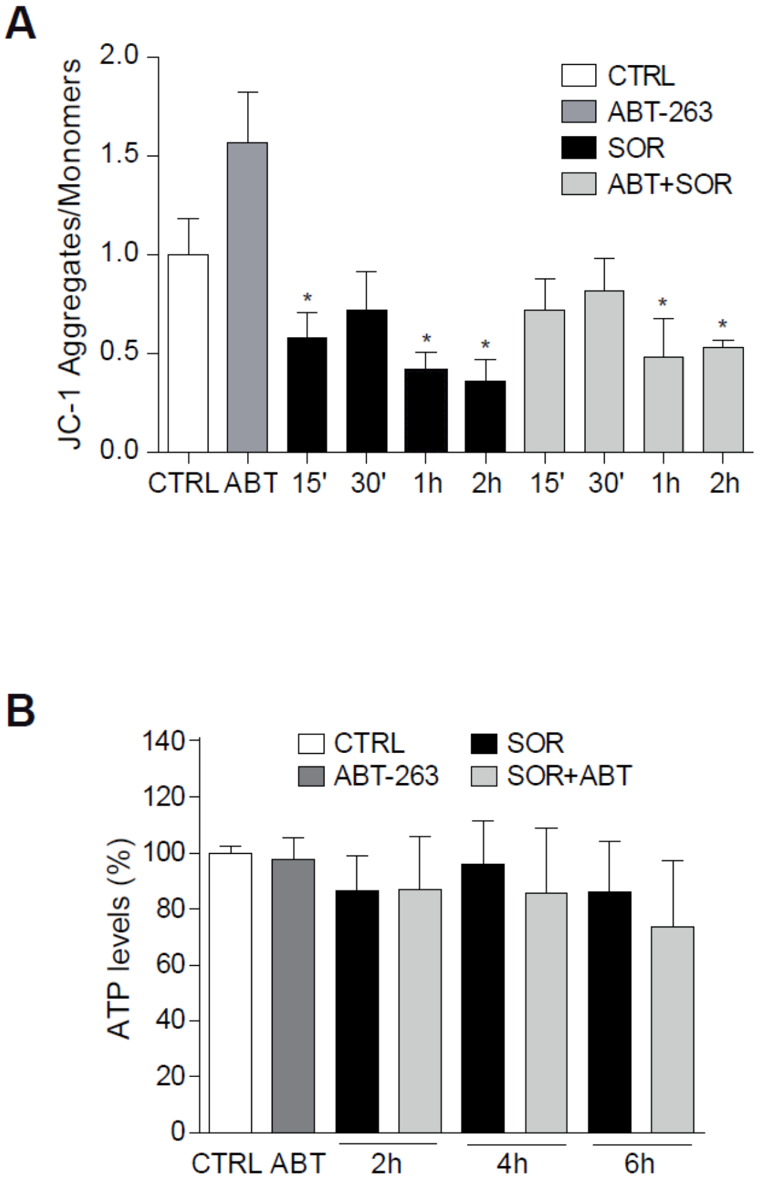

Supplementary Figure 7: Effect of sorafenib and navitoclax on MMP and ATP levels. Hep3B cells were exposed to sorafenib (10 μM) with or without ABT-263 (5 μM). **(A)** Mitochondrial membrane potential by JC1 determination and **(B)** ATP levels were measured. (n=3).*P< 0.05 vs. control Hep3B cells.

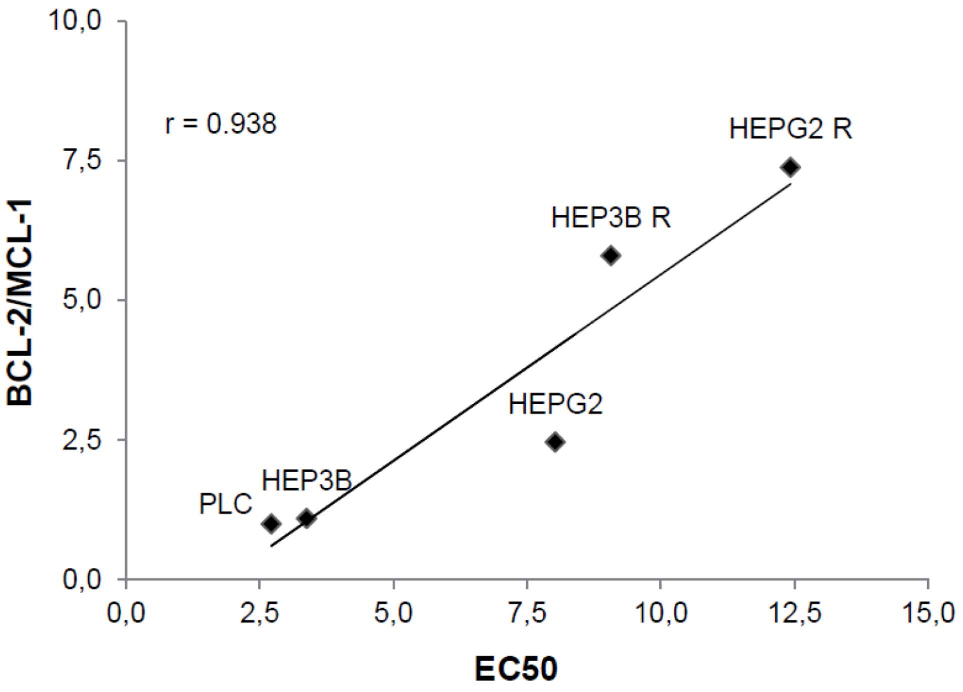

Supplementary Figure 8: Correlation between relative BCL-2/MCL-1 ratio and sorafenib cytotoxicity in hepatoma cell lines. mRNA levels of BCL-2 and MCL-1 were measured by qPCR and MTT assays were performed after 24 h of sorafenib exposure.
